# Incidental Abdominal Pseudocyst in a Patient on Peritoneal Dialysis: A Case Report

**DOI:** 10.7759/cureus.88008

**Published:** 2025-07-15

**Authors:** Cesar Emmanuel Vazquez Padilla, Jose Maria Zepeda Torres, Carolina Topete Rodríguez, Andrea Michelle Salas Carlock, Saul Vidrio Capacete, Sergio Flores González

**Affiliations:** 1 Surgery, Mexican Social Security Institute, General Zone Hospital No. 89, Guadalajara, MEX; 2 Surgery, Mexican Social Security Institute, Specialty Hospital, National Medical Center of the West, Guadalajara, MEX; 3 Surgery, Mexican Social Security Institute, General Zone Hospital 1, Tepic, MEX; 4 General Surgery, Social Security Institute, Specialty Hospital, National Medical Center of the West, Guadalajara, MEX; 5 Bariatric and Metabolic Surgery, Hospital Civil de Guadalajara Dr Juan I Menchaca, Guadalajara, MEX

**Keywords:** abdominal pseudocyst, incidental diagnosis, peritoneal dialysis complications, peritoneal dialysis (pd), ct

## Abstract

We report an intriguing case of an incidental abdominal pseudocyst in a patient undergoing long-term peritoneal dialysis. Although pseudocysts are rare, they represent a significant complication in peritoneal dialysis patients, often arising secondary to chronic peritoneal inflammation, peritonitis episodes, or catheter-related factors. In this case, the pseudocyst was an incidental finding on imaging, with no immediate symptoms but potential implications for dialysis efficacy and future intra-abdominal complications. The case underscores the importance of vigilant imaging assessment and multidisciplinary management to monitor such lesions and prevent progression to more serious issues, including infection or loss of dialysis access. Recognizing these pseudocysts is critical for optimizing ongoing management and preserving peritoneal dialysis as a feasible treatment modality.

## Introduction

An incidental abdominal pseudocyst in a patient undergoing peritoneal dialysis is a rare but clinically significant finding. Abdominal pseudocysts in the context of peritoneal dialysis are typically acquired, non-epithelial-lined fluid collections that develop as a complication of chronic peritoneal inflammation, often following episodes of peritonitis or prolonged exposure to dialysis solutions. These pseudocysts can range from asymptomatic incidental findings to causes of abdominal discomfort, impaired dialysis efficacy, or intra-abdominal complications requiring intervention. The identification and management of pseudocysts are crucial in preventing more severe sequelae such as infection or rupture [1-3]. We present an interesting case of an incidental abdominal pseudocyst in a patient undergoing peritoneal dialysis (PD), highlighting its clinical significance and management implications.

## Case presentation

A 30-year-old male with chronic renal failure presented with respiratory difficulty, bilateral lower limb edema (+++), and cough. He reported that his symptoms began 15 days prior with intermittent productive cough, no fever, minimal dyspnea on exertion, and occasional nausea. Over the past week, his condition worsened, with four episodes of vomiting and increased shortness of breath on moderate effort. While awaiting an outpatient appointment, he experienced an acute episode of respiratory distress, muscle weakness, and nausea without vomiting, prompting hospital admission to the nephrology service. Physical examination revealed a conscious, oriented, and cooperative patient with mild pallor, no cranial exostoses; symmetrical eyes with isocoric, normally reactive pupils; slightly dehydrated oral mucosa; and a cylindrical neck without jugular venous distension, a central, movable trachea, no nuchal rigidity, adenopathies, or palpable masses. His thorax showed normal respiratory movements, but auscultation revealed decreased breath sounds at the left lung base, with fine inspiratory crackles. Abdominal examination disclosed a soft, depressible, semiglobose abdomen without tenderness or signs of peritonitis; notably, a peritoneal dialysis catheter was visible in the right flank. His upper limbs appeared normal with preserved strength and adequate capillary refill in 3 seconds, while the lower limbs exhibited slightly delayed capillary refill, preserved strength, and marked edema (+++). Laboratory tests are shown in Table 1.

**Table 1:** Laboratory tests

Laboratory Test	Result	Reference range
Leukocytes	17,100 /μL	4,500 - 11,000 /μL
Neutrophils (%)	91%	40% - 60%
Hemoglobin	11.2 g/dL	13.8 - 17.2 g/dL (men); 12.1 - 15.1 g/dL (women)
Platelets	215,000 /μL	150,000 - 450,000 /μL
Glucose	97 mg/dL	70 - 99 mg/dL
Creatinine	0.5 mg/dL	0.6 - 1.2 mg/dL
Blood Urea Nitrogen (BUN)	21 mg/dL	7 - 20 mg/dL
Prothrombin Time	11.9 seconds	11 - 13 seconds
INR	0.9	0.8 - 1.1
Total Bilirubin	7.16 mg/dL	0.1 - 1.2 mg/dL
Direct Bilirubin	4.6 mg/dL	0.0 - 0.3 mg/dL
Indirect Bilirubin	2.4 mg/dL	0.1 - 1.0 mg/dL
AST (Aspartate Aminotransferase)	68 U/L	10 - 40 U/L
ALT (Alanine Aminotransferase)	109 U/L	7 - 56 U/L
Alkaline Phosphatase	767 U/L	44 - 147 U/L
GGT (Gamma-Glutamyl Transferase)	438 U/L	8 - 61 U/L

**Figure 1:**
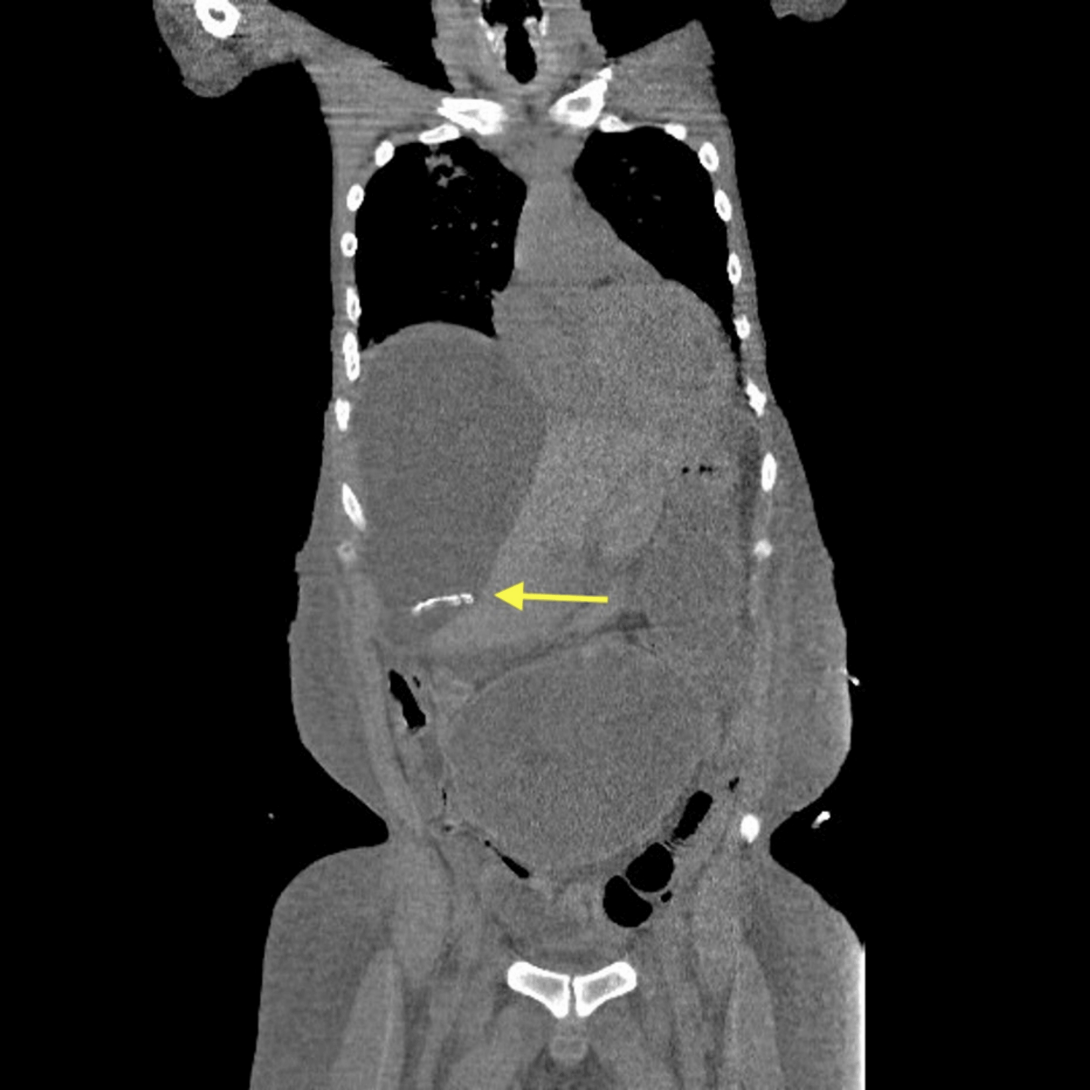
Dialysis catheter tip in the right flank and revealed a well-defined, fluid-filled cystic lesion consistent with an inclusion cyst (coronal view)

**Figure 2:**
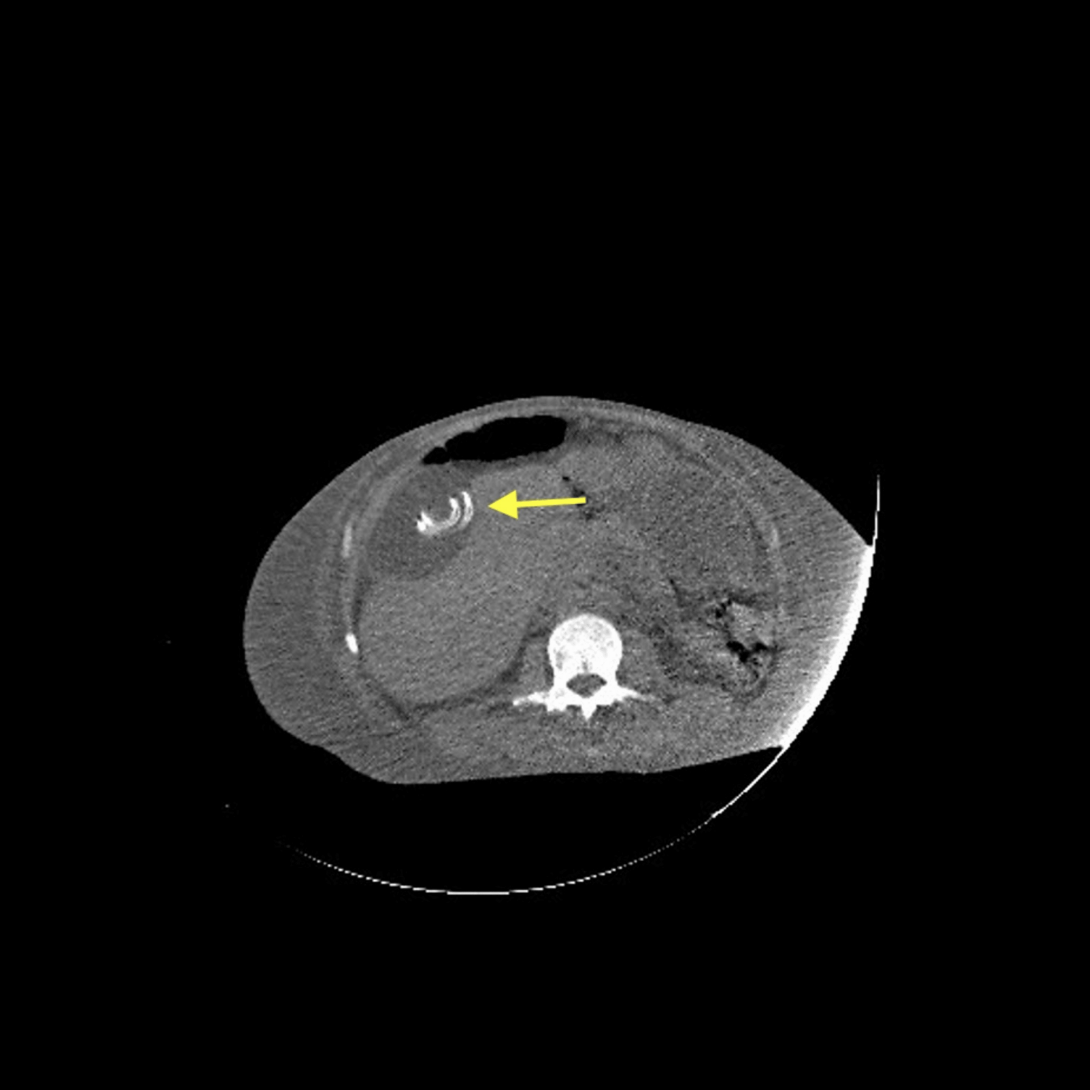
Dialysis catheter tip in the right flank and revealed a well-defined, fluid-filled cystic lesion consistent with an inclusion cyst (axial view)

**Figure 3:**
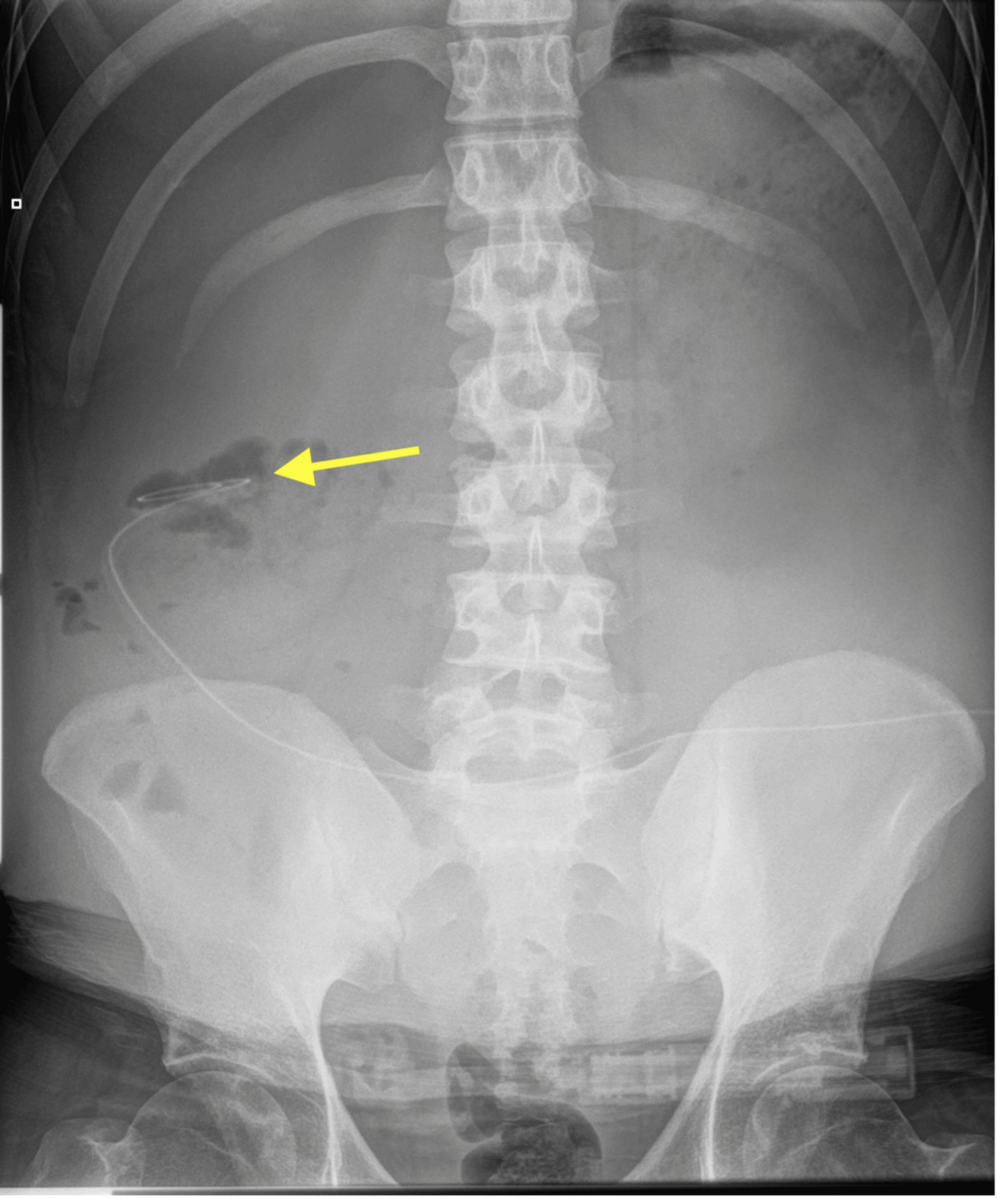
Abdominal X-ray showing the catheter tip in the right flank

Imaging on 25/05/2025 demonstrated the dialysis catheter tip located in the right flank, along with a well-defined, fluid-filled cystic lesion consistent with an inclusion cyst (Figures 1, 2). An abdominal X-ray further confirmed the catheter position in the right flank (Figure 3). The patient's presentation with respiratory symptoms prompted assessment, revealing an incidental inclusion cyst that, although asymptomatic, could have implications for future management. Throughout his hospitalization, the patient was closely monitored, maintaining fluid balance via peritoneal dialysis with a net negative fluid removal of 13 liters. He experienced two hypertensive emergencies requiring intravenous antihypertensives, followed by episodes of fever and increased oxygen requirements. The incidental cyst raised considerations about its nature and potential future complications in the context of renal treatment. His clinical course was supportive, including antibiotics for pneumonia and careful observation of the cyst’s evolution, which remained asymptomatic.

## Discussion

Several key aspects of the significance of pseudocyst

Potential Complications of PD

Pseudocyst formation is an uncommon but recognized complication of PD, often associated with prior or ongoing peritonitis, catheter-related infection, or chronic inflammatory changes in the peritoneum. These cysts can develop during active PD or, less commonly, after discontinuation of therapy [1-3].

Clinical Implications

While some pseudocysts may be asymptomatic and discovered incidentally, they can cause progressive difficulty with dialysate inflow or outflow, leading to decreased solute clearance and ultrafiltration failure. In some cases, they may present with abdominal pain, distension, or signs mimicking encapsulating peritoneal sclerosis or neoplastic processes [1-3].

Abdominal pseudocysts are a rare but recognized complication in patients undergoing peritoneal dialysis, typically arising in the context of prior or ongoing peritonitis or catheter-related issues. The potential complications associated with these pseudocysts include the following.

Recurrent or persistent abdominal symptoms: Patients may experience abdominal pain, distension, and discomfort due to the mass effect of the pseudocyst or impaired dialysate flow. These symptoms can be persistent and may recur even after initial drainage attempts, as highlighted in case reports of patients with recurrent symptoms despite intervention [1,2].

Impaired dialysis efficacy: Pseudocyst formation can lead to progressive difficulty with instillation and drainage of peritoneal dialysis fluid, resulting in decreased solute clearance and loss of ultrafiltration. This may necessitate a switch from peritoneal dialysis to hemodialysis if the pseudocyst cannot be adequately managed [2].

Infection: Pseudocysts may become secondarily infected, particularly in the setting of prior peritonitis, leading to intra-abdominal sepsis. This risk is underscored by the association between pseudocyst formation and previous episodes of peritonitis in reported cases [1,2].

Diagnostic confusion and delay: Imaging findings of pseudocysts can mimic neoplastic processes, leading to diagnostic uncertainty and potentially delayed or inappropriate management. This necessitates careful evaluation, often with surgical exploration or biopsy to exclude malignancy [4,5].

Need for surgical intervention: Persistent or complicated pseudocysts may require surgical management such as cyst excision or marsupialization. Surgical intervention carries its own risks, including bleeding, infection, and postoperative complications [1].

Loss of peritoneal dialysis access: In cases where the pseudocyst involves or encloses the catheter tip, catheter removal may be necessary, resulting in the loss of peritoneal dialysis as a modality [2].

While the literature does not report a high incidence of life-threatening complications, such as hemorrhage or bowel obstruction, specifically in the context of dialysis-related pseudocysts, the potential for significant morbidity exists, particularly if infection or loss of dialysis access occurs. Close follow-up and a multidisciplinary approach are essential for optimal management [1,2].

Diagnostic Considerations

Imaging (ultrasound or CT) typically reveals a well-circumscribed fluid collection, sometimes encasing the PD catheter tip. Differentiation from other cystic lesions (e.g., neoplastic cysts, mesenteric cysts, or pancreatic pseudocysts) is essential, as management strategies differ. Histology, when available, usually shows a non-epithelialized, inflammatory wall, distinct from neoplastic or true mesothelial cysts [1-3].

Management and Prognosis

Asymptomatic pseudocysts may be monitored, but symptomatic or complicated cases often require intervention such as drainage, catheter removal, or surgical excision. Persistent or recurrent pseudocysts may necessitate transition to hemodialysis, especially if PD function is compromised or infection risk is high [1-3].

Surveillance and Follow-Up

The presence of a pseudocyst warrants close clinical and imaging follow-up, given the potential for recurrence, infection, or progression to more severe complications such as encapsulating peritoneal sclerosis [1,3].

## Conclusions

This case underscores the importance of recognizing incidental abdominal pseudocysts in patients undergoing peritoneal dialysis, as these lesions, although often asymptomatic, can have substantial clinical implications. While pseudocyst formation is a rare complication typically associated with prior peritoneal inflammation or catheter-related issues, its presence can compromise dialysis efficacy, cause persistent abdominal symptoms, and predispose to infections or other intra-abdominal complications. Early identification through imaging allows for careful monitoring and tailored management, including potential intervention if the cyst becomes symptomatic or complicated. This case highlights the necessity of a multidisciplinary approach and careful follow-up to prevent adverse outcomes. Further research is warranted to better understand the pathogenesis, optimal management strategies, and long-term prognosis of pseudocysts in PD patients, ultimately improving patient care and preserving the modality of peritoneal dialysis whenever possible.
